# Participating in a School-Integrated Daily Exercise Program Improves Motor Performance Significantly in School-Children

**DOI:** 10.3390/ijerph20064764

**Published:** 2023-03-08

**Authors:** Denise Homeyer, Nima Memaran, Momme Kück, Lena Grams, Jeannine von der Born, Elena Bauer, Martina Schwalba, Arno Kerling, Nadine von Maltzahn, Alexander Albrecht, Axel Haverich, Meike Stiesch, Anette Melk, Uwe Tegtbur

**Affiliations:** 1 Department of Rehabilitation and Sports Medicine, Hannover Medical School, 30625 Hannover, Germany; 2 Department of Pediatric Kidney, Liver and Metabolic Diseases, Hannover Medical School, 30625 Hannover, Germany; memarandadgar.nima@mh-hannover.de (N.M.);; 3 Department for Dental Prosthodontics and Biomedical Materials Science, Center for Dentistry and Oral Medicine, Hannover Medical School, 30625 Hannover, Germany; 4 Department of Cardiothoracic, Transplantation, and Vascular Surgery, Hannover Medical School, 30625 Hannover, Germany

**Keywords:** physical activity, motor skill, exercise, ergometry

## Abstract

Children’s sedentary time has increased, while daily physical activity and motor performance have decreased. We evaluated an integrated school-based exercise program by assessing changes in motor skills after one year and comparing these changes to children who did not participate. We included 303 children from five schools in this longitudinal study and assigned them either to the exercise group (EG; n = 183 with daily exercise program) or the waiting group (WG; n = 120). Motor skills were assessed at baseline and after one year. Mixed modeling was used to analyze inter-group differences of change in motor skills and to determine the effect of sex, age group, and weight status. EG improved more strongly than WG for sprint, side jumps (both *p* = 0.017), stand and reach (*p* = 0.012), and ergometry (*p* ≤ 0.001) when compared to WG. Girls improved more strongly in the sit-ups than boys, second graders more than fifth graders in the backwards balance and the ergometry, and non-overweight children more in the standing long jump than overweight children. The exercise program is effective in increasing motor skills and physical fitness. Girls were not disadvantaged, and overweight children profited as much as their non-overweight peers in all categories but one.

## 1. Introduction

Children and adolescents do not exercise sufficiently [1,2]. The target level of 60 min physical activity per day recommended by the World Health Organization (WHO) [3] is only reached by 22.4% of girls and 29.4% of boys aged 3–17 years in Germany and is less likely to be reached with increasing age [2]. This lack of physical activity negatively affects overall health and physical capability in children [4,5] and can be both a cause and consequence of being overweight [6,7]. Likewise, sedentary time, associated with poorer motor coordination [8], has increased for children, especially during school [9]. In parallel, motor performance is low [10,11]. Between 1975 and 2002, motor performance of German children and adolescents decreased by 10% [12], and was interestingly more pronounced in the 12- to 17-year-olds with 12.5% than the 6- to 11-year-olds with 5.5% [12], demonstrating a clear role of age. Furthermore, motor performance is dependent on weight status as well as sex. For instance, overweight and obese children exhibited impaired motor performance in 43.4% to 70.8%, respectively, and show poorer results in dynamic body coordination [13]; girls exhibited higher performance in fine motor skills, while boys showed higher performance in catch and dribble gross motor skills [14,15].

The downward trend in motor performance persisted until the turn of the century and seems to have stabilized for Germany [16] and internationally [17], with no further decrease in motor skills. Working towards increasing children’s motor skills again is vital, as they are essential for healthy and appropriate development [18]. Most importantly, low motor performance in childhood is associated with an increased risk of cardiovascular disease in adulthood [19,20]. The value of health interventions during childhood is, therefore, evident.

Children spend much time in school, mostly seated, leading to lower daily physical activity [21]. Therefore, interrupting sedentary periods by integrating several brief activities over the school day can significantly increase daily physical activity. Such a lifestyle modification is as effective as a structured exercise program, improving cardiorespiratory fitness [22] and motor skills [23].

Several studies have described the effect of a school-based physical activity program on improving motor skills. They, however, may show particular disadvantages. Some can often only be implemented with a professional physical activity teacher [24,25,26], have a short intervention duration [24,27], or only offer extracurricular activities [25,28,29], which may lead to predominantly targeting children that already show a certain motivation for exercise. Others, such as the One Mile program, successfully increased daily physical activity with a practical, low-cost in-school program that improved endurance and skinfold thickness [30] but did not investigate the program’s effect on motor skills.

Our study aimed to develop and implement a comprehensive exercise program that can effectively improve a variety of motor skills; a program that is tailored to children that is simple, inexpensive, and can easily be integrated into the school setting; can be conducted with little additional staff beyond the initial phase; and takes place during the regular curriculum, so that all children have the opportunity to participate.

We further aimed to evaluate this program with children from two different age groups by assessing changes in motor skills after one year and comparing these changes to children who did not participate.

## 2. Materials and Methods

### 2.1. Study Design

A convenience sample of primary and secondary schools in Lower Saxony and North Rhine-Westphalia was approached. Of those, five schools agreed to participate. The 2nd and 5th grades were chosen because 4th-grade children would have been lost to follow-up as they changed from primary to secondary school. The 1st grade was not chosen as it was their first year in school. Therefore, this longitudinal cohort study investigated children from all 2nd-grade and 5th-grade classes (aged 8.2 ± 0.5 and 11.4 ± 0.5, respectively) after obtaining written informed consent from the parents or caregivers and assent from the participating children. The baseline examination was performed between April and June 2017 (v0), and the follow-up examination was one year afterward (v1). The assignments to the respective groups could not occur on an individual level but per class. Therefore, after completing v0, classes were randomly assigned to the exercise program (i.e., the exercise group, EG) or made to wait one year before starting with the exercise program (i.e., the waiting group, WG). Every participating grade of each school contained WG as well as EG classes in order to assure comparability. This study is in line with the principles of the Declaration of Helsinki. Approval was granted by the Ethics Committee of the Hannover Medical School (No. 7290).

### 2.2. Motor Tests

For the comprehensive motor skill assessment, we chose the German Motor Test 6–18 (Deutscher Motorik-Test 6–18, DMT 6–18), an established tool to assess motor skills in children that is recommended by the German Society of Sport Science [31]. This test battery has established high objectivity, reliability, and validity for evaluating motor skills in children and adolescents aged 4–17 [31]. A detailed description for each test item is given in the supplementary material. In short, speed was assessed through a 20 m sprint (measured in seconds), coordination in precision-tasks by backwards balancing (measured in the number of steps), coordination under time pressure with side jumps (measured in the number of jumps), trunk and sciatocrural muscle group flexibility through the stand and reach test (measured in centimeter), endurance-strength of the upper extremities and trunk muscles with push-ups (measured in the number of push-ups), endurance-strength of the torso with sit-ups (measured in the number of sit-ups), and lower extremity jumping power with standing long jumps (measured in centimeter). All assessors were uniformly trained by an experienced sports scientist (D.H.) to ensure the accuracy of the measurements. Every assessment of motor skills (i.e., at v0 and v1) was conducted under the supervision of the same experienced sports scientist (D.H.).

### 2.3. Ergometry

Maximum endurance capacity was assessed via bicycle ergometry according to a modified Godfrey protocol [32], with maximum Watt per kg bodyweight (W/kg) as the endpoint. The workload was increased in 15 Watt steps at 1-min intervals. All subjects were encouraged to exercise until exhaustion (breathlessness and leg muscle pain or a maximum calculated heart rate (calculated 220—age in years) [33], multiplied by 0.85 to account for potential overestimation of the formula in children [34]).

### 2.4. Exercise Program

The exercise program was designed to be primarily conducted by teachers, who were guided by sports scientists who visited every class on two days every week for the initial phase. It comprised of several modules. The main module was the exercise impulses, where children performed a 5-min exercise during every single lesson, coordinated by the teachers (5–6 lessons per day, 5 days per week; identical within a school grade for both groups). Exercises were chosen from the literature [35,36] to be specifically suitable and fun for children, take a maximum of 5 min, and easily be performed in the classroom. They represent four categories (strength, endurance, coordination, and relaxation), with 10 different exercises for every category. Since speed and flexibility were aspects in almost all exercises, they were not assigned separate groups. Most of the exercises were related to a school subject to facilitate integration into the classroom (e.g., “math jogging”, where children jump and squat depending on the result of the calculation). EG classes were provided with a box of index cards describing every exercise. A list and short description of every exercise are given in the supplemental material, and an example of the cards in Figure S1. Teachers received detailed instructions during an evening workshop by trained sports scientists on how to perform these exercises correctly and to use all four categories evenly.

In addition to the movement impulses, the following modules were provided once per week, under instructions from a sports scientist. A 15–20 min early morning exercise module was offered before the first lesson; a 15-min exercise module was provided during the great break and a 45-min module after school or before afternoon classes for the secondary school students. Once per week, an afternoon module of 45 min was organized for every class. Lastly, teachers and children from the EG received general information on the benefits of daily physical activity before the start of the program. Teachers were furthermore instructed to document each exercise impulse and the additional modules.

### 2.5. Anthropometric Measurements

Body height and body weight were measured according to Dippelhofer et al. [37] and used to calculate the body mass index (BMI). BMI was normalized for age and sex, using WHO normative data [38] and expressed as z-scores, with a z-score of 0.0 representing the 50th percentile. Children were grouped according to BMI into non-overweight (defined as BMI < 85th percentile, i.e., z-score < 1.036) or overweight (defined as BMI ≥ 85th percentile, i.e., z-score ≥ 1.036) [39].

### 2.6. Statistical Analysis

All calculations were performed using Statistical Analysis Software Enterprise Guide 7.1 (Cary, NC, USA) and SPSS (Version 28, IBM corp., Armonk, NY, USA). Categorical variables are given as frequencies and percentages, and continuous variables as means and standard deviations. The change in motor test results was the primary endpoint. For the univariate analyses at baseline, the two-sided t-test was used for continuous variables and the χ^2^-test for categorical variables. For the univariate analysis, a paired *t*-test was used for within-group comparisons. The interaction between time and group was calculated with an ANOVA with repeated measurements for the between-group comparison. As sex, age, and weight status are known to influence motor skills, linear regression modeling was utilized to calculate corrected means for the change in motor skills (calculated as the Delta (Δ) of the result of v1 − v0) adjusted for sex, age, and BMI, differentiated by the WG and EG group category. For the analysis of interdependent factors, we calculated a separate multivariable linear regression model for every endpoint, each with the group category and interaction terms between (i) the group category and sex, (ii) the group category and age group, and (iii) the group category and BMI category, as covariates. A *p*-value of <0.05 was considered significant.

## 3. Results

### 3.1. Study Population

Out of 598 children that were invited, 357 (n = 205 for second grade, n = 152 for fifth grade) agreed to participate and were included in the study. The whole cohort of 357 children is described in detail [40]. Out of these, 303 children completed one or more of the motor skill tests at both time points v0 and v1 and comprise the sample for this study (EG n = 183; WG n = 120; Figure S2). From these 54 dropouts, 13 children were absent from school during the day of examination due to illness, 22 had left the school, and 19 declined to participate further. Baseline characteristics are given in Table 1. At baseline, there were no significant differences between the EG and the WG regarding gender, age, weight, height, or BMI.

### 3.2. Motor Skills at Baseline

At baseline, there were no significant differences in the motor skills between the WG and the EG, except for the push-ups, where the EG achieved more repetitions (WG 12.1 ± 3.3 repetitions, EG 13.2 ± 3.9 repetitions, *p* = 0.026; Table 1). Boys performed better in the sprint, stand and reach, sit-ups, standing long jumps, and ergometry than girls, with no difference for the backwards balance, side jumps, and push-ups (Table S1). Fifth graders were better in the sprint, side jumps, and standing long jump, and second graders were better in the stand and reach test (Table S2). Compared to the overweight children, non-overweight children performed better in all tests except for the stand and reach test, where no significant difference could be seen between the two groups (Table S3).

### 3.3. Physical Activity Due to the Exercise Program

As a measure of program fidelity, teachers were tasked to document when performing an exercise impulse during the lesson and to document participation in the additional modules of the exercise program. Based on this documentation, an average of 24 min and 34 min of additional exercise per day due to participation in the total exercise program could be achieved for the second and fifth graders, respectively. This calculation did not include regular curricular physical education classes that were available to EG and WG.

### 3.4. Within-Group Change in Motor Skills after One Year

The assessments of motor skills were one year apart (time between v0 and v1 for EG 370 ± 22 days; WG 367 ± 19 days, *p* = 0.235). When looking at within-group changes after one year, the EG showed significant improvements in all motor skill test items. The WG revealed significant improvement for the sprint, backwards balance, side jumps, push-ups, and the standing long jump, but not in the stand and reach, sit-ups, or ergometry (Table 2).

### 3.5. Between-Group Change in Motor Skills after One Year

After one year, the unadjusted between-group analysis of the change showed that EG children improved significantly more strongly in the sprint, backwards balance, side jumps, stand and reach, sit-ups, and ergometry, than the WG children (Table 2).

**Figure 1:**
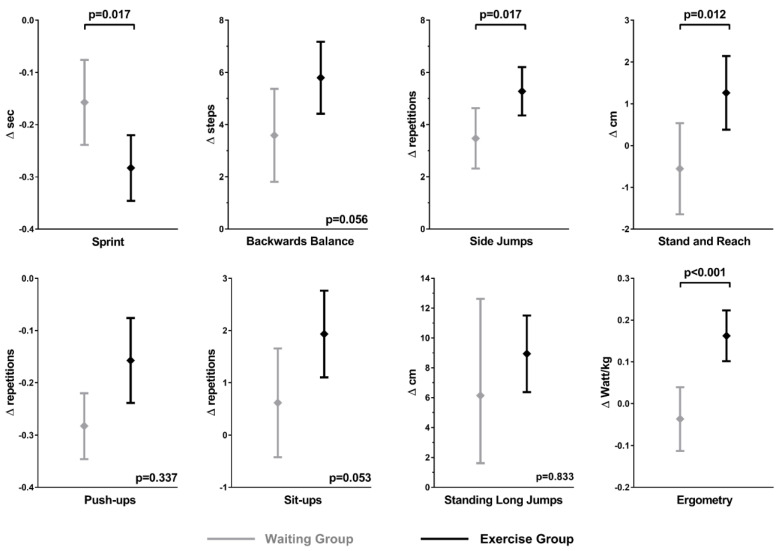
Comparison of the corrected means of the change in motor skills after one year between the waiting group (grey) and the exercise group (black), adjusted for age, sex, and BMI. The exercise group improved significantly more strongly than the waiting group in the sprint, side jumps, stand and reach, and ergometry test items and showed a tendency for stronger improvement in the backwards balance and sit-ups, while no significantly different change could be observed for the standing long jumps and push-ups.

After adjusting for sex, age, and BMI, the EG still exhibited a significantly stronger improvement in the sprint, side jumps (both *p* = 0.017), stand and reach (*p* = 0.012), and ergometry (*p* < 0.001), with a tendency for backwards balance and sit-ups (*p* = 0.056 and *p* = 0.053, respectively; Figure 1).

### 3.6. Interdependent Factors Associated with the Change in Motor Skills

In order to analyze whether the exercise program affected children differently according to their sex, age group, or BMI category, separate linear regression models were calculated for each endpoint, with the change in the motor skill test item as the dependent variable and interaction terms between group category and sex, group category and age group, or group category and BMI category, as covariates. The complete models are given in Table S4a–h. Table 3 summarizes the results of these separate models. WG boys increased the number of push-ups more than WG girls, and EG girls expanded the number of sit-ups more greatly than EG boys. Second-grade WG children improved significantly more strongly than fifth-grade WG children in the sprint and push-ups, and second-grade EG children improved significantly more strongly than EG fifth graders in the backwards balance, push-ups, and ergometry. Regarding the BMI category, non-overweight EG children improved the standing long jump more robustly than their overweight EG peers.

## 4. Discussion

This prospective, longitudinal cohort study demonstrates an effective school-based exercise program that can be easily implemented into school life and significantly improves children’s motor skills after one year compared to peers who did not participate.

As school is a socialization institution that is a well-suited setting for programs intending to positively influence healthy behavior [41], it is ideal for this inclusive and effective exercise program. Furthermore, a school-based program is easily accessible for all children, regardless of their socioeconomic status or intrinsic motivation to be physically active in their free time.

Our exercise program shows particular strengths. Its simplicity is particularly noteworthy, as this is the prerequisite for long-term implementation, in contrast to other programs requiring additional equipment [26] or staff [28,29]. We only require the index card box and no additional space or venues. Teachers can effectively conduct it after the initial phase. The exercise program was implemented and carried out for one year. The longer duration of the intervention compared to other studies [25,27], allowed analysis of the long-term effects. Lastly, the program requires minimal additional time and does not disrupt the curriculum [26,28,29]. These characteristics make it easily implementable in different school settings.

A major strength of the study is the objective assessment of the effect of participating in the exercise program on a variety of motor skills and comparing the results with those of age-matched peers from the same school who did not participate. The comparison of the change in motor skills between the EG and WG enabled us to concisely discern the impact of the exercise program from potential confounders, such as physiological development, or a potential learning effect from repeatedly performing these motor tests. By engaging in the exercise program for one year, children saw a clear improvement in their speed, coordination under time pressure, and flexibility (measured by the sprint, side jumps, stand and reach tests, respectively), as well as their maximum endurance capacity (gauged bicycle ergometry). These advancements were significantly stronger in the EG than the WG and remained significant after adjusting for age, sex, and BMI. Moreover, a tendency could be observed for coordination in precision-tasks and endurance-strength of the torso (showed by the backwards balance and sit-ups, respectively).

Apart from the apparent reason for the observed effects, i.e., the additional physical activity in the EG, another factor may also play a role. It can be speculated that participation in daily exercise and performing fun movement impulses during every lesson every day for the intervention period of one year leads to a higher personal affinity to exercise and, therefore, being physically more active outside the school setting as well [42]. It would be especially desirable if this effect could be achieved with children that would otherwise not have considered sports and exercise a leisure activity, thereby creating a positive trajectory towards a healthier lifestyle. To address this aspect, we are currently enrolling a study with a larger sample and quantifying 24 h of physical activity to gauge the change in daily activity after school.

Of note, upper extremity strength and lower extremity jumping power (tested through push-ups and standing long jumps, respectively) did not show a differing development between the groups. This result could be due to the fact that building additional strength, such as jumping power, may require a longer intervention period and higher intensity [43]. Upper extremity strength and, to a lesser degree, lower extremity jumping power could not be targeted as intensely by the exercise impulses during lessons, which constituted the main module of the exercise program. Possible reasons may be that in order to generate an adequate stimulus for muscle growth, the use of weights or a higher number of repetitions and sets would be necessary and would require instructions on the proper exercise technique in order to prevent injuries (which could compromise the game character of the exercise). Therefore, it is possible that the exercise program may not have been intense enough to boost these specific motor skills significantly. In addition, these two tests include complex movement sequences (e.g., the timing of the arm swing and jump), which might require more targeted training.

At baseline, we saw differences between the boys’ and girls’ results, which align with the literature [44]. However, the results were similar for the side jumps and push-ups, whereas other studies saw sex differences as well [45,46]. When investigating the differential effect of the program between the sexes, our data showed that the increase in push-ups was less for girls than boys in the WG. Interestingly, this effect could not be observed in the EG, suggesting that the exercise program helped the girls keep up with the boys. With the sit-ups, EG girls profited significantly more than EG boys. Considering that no sex disadvantage for EG girls was seen for the push-ups and even an advantage for EG girls for sit-ups, it seems that our program led to girls benefiting more in building strength-endurance than boys.

The program was more effective for second graders in the backwards balance and ergometry. Second graders increased the number of push-ups more strongly than fifth graders, but this was seen in both WG and EG. Taken together with the fact that WG and EG were not significantly different in this particular test item, it may be that the observed effect is due to physiological development in this age group [47].

It was not surprising to find that overweight children performed worse than their non-overweight peers in all the tested motor skill categories (except for flexibility), in line with the literature [48,49]. Overweight children are known to show less development in their motor skills [50]. It was, therefore, surprising to see that the change in the tested motor skills in overweight children did not significantly differ from that in their non-overweight peers in any test item other than the standing long jump. In particular, our data showed no disadvantage for overweight children for the change in ergometry. This result is especially important, as high physical fitness alleviates the metabolic risk of being overweight [51,52]. Even though we could not see a significant catch-up development (i.e., a stronger effect in overweight than non-overweight children), this finding still is very encouraging, as it suggests that overweight children can keep up with their non-overweight peers at this age if they are physically active. Overweight children are more likely to originate from socially disadvantaged backgrounds [53,54], associated with lack of exercise [55,56]. Furthermore, overweight children are more likely to be reluctant to engage in sports activities because of shame or the fear of being bullied [57]. Placing this easily accessible exercise program into the school setting free of charge can represent a suitable tool to reach disadvantaged groups that may not be able to engage in extracurricular sports due to financial opportunities, lack of intrinsic motivation, or may be reluctant to do so.

Our study shows several strengths. It is a multi-site prospective longitudinal study on a large cohort of children from two age groups. Secondly, we employed a standardized protocol of motor tests that are suitable for children conducted by uniformly trained sports scientists. Thirdly, comparing the change in motor skills of children to that of age-matched peers from the same schools allowed us to discern the exercise program’s effect precisely. The study also carries a few limitations. Based on the modules’ documentation, we could not reach the intended goal of an additional 60 min of daily activity. One reason was partially incomplete documentation of exercise impulses they conducted during the lessons by teachers. It can, therefore, be assumed that the actual average exercise time was higher than reported. We intended to use accelerometers as a measure of daily physical activity and a measure of fidelity to the program. We could not achieve this because many devices were not returned or misused. These data would have allowed us to not only measure changes in overall physical activity but also to account for differences in physical activity outside of school, especially during the holidays. However, as WG and EG children attend the same school and are of the same age, it can be assumed that both groups share comparable social and financial backgrounds, live in the same area, and have comparable leisure habits. Therefore, the possibility to compare the EG to the WG is a particular strength of the study that allowed us to demonstrate the beneficial effect of the exercise program on the participants’ motor skills and physical fitness. A stringent and facile measure to document intervention fidelity is needed to capture all measures taken in future studies fully. Our results are limited to the two age groups of second-grade and fifth-grade children; therefore, generalization to other age groups is limited. We cannot, therefore, make valid inferences on trajectories of motor development in children across different age groups. Further studies involving other age groups, especially teenagers, are warranted.

## 5. Conclusions

In conclusion, our study presents a simple and effective in-school exercise program that can be easily implemented into the daily school routine, significantly increasing motor skills and physical fitness.

## Figures and Tables

**Table 1:** Baseline characteristics of the exercise group and waiting group.

	WG		EG		*p*
	Mean ± SD or Number (%)	n	Mean ± SD or Number (%)	n	
Sex (m/f)	68/52	120	94/89	183	0.366
Age (years)	9.7 ± 1.7	120	9.4 ± 1.6	183	0.141
Height (cm)	140.4 ± 12.1	119	138.8 ± 10.9	183	0.223
Weight (kg)	37.4 ± 12.4	119	35.3 ± 11.5	183	0.129
BMI z-score	0.40 ± 1.07	119	0.22 ± 1.15	183	0.178
Overweight children	32 (27%)	119	48 (26%)	183	0.899
Sprint (sec)	4.5 ± 0.5	109	4.6 ± 0.5	173	0.196
Backwards Balance (steps)	29.4 ± 9.5	102	28.9 ± 10.4	159	0.709
Side Jumps (repetitions)	26.9 ± 5.7	108	25.3 ± 7.8	164	0.056
Stand and reach (cm)	−1.1 ± 6.3	97	−0.5 ± 7.5	144	0.472
Push-ups (repetitions)	12.1 ± 3.3	103	13.2 ± 3.9	152	0.026
Sit-ups (repetitions)	19.3 ± 6.0	112	18.6 ± 6.2	169	0.405
Standing long jump (cm)	128.1 ± 21.9	112	127.7 ± 26.0	170	0.895
Ergometry (W/kg)	3.1 ± 0.7	117	3.1 ± 0.7	178	0.671

Abbreviations: EG, exercise group; SD, standard deviation; WG, waiting group. *p*-values < 0.05 are bolded.

**Table 2:** Univariate within- and between-group analysis of the change in motor skills after one year.

	WG				EG				ANOVA	
	v0	v1			v0	v1			Time	Time × Group
	Mean ± SD	Mean ± SD	n	*p*	Mean ± SD	Mean ± SD	n	*p*	*p*	*p*
Sprint (sec)	4.5 ± 0.4	4.3 ± 0.4	104	**<0.001**	4.6 ± 0.5	4.3 ± 0.4	169	**<0.001**	**<0.001**	**0.017**
Backwards Balance (steps)	29.4 ± 9.6	32.9 ± 10.3	96	**<0.001**	28.8 ± 10.6	34.7 ± 9.6	155	**<0.001**	**<0.001**	**0.031**
Side Jumps (repetitions)	26.7 ± 5,6	30.2 ± 5.6	102	**<0.001**	25.5 ± 7.8	30.8 ± 8.4	155	**<0.001**	**<0.001**	**0.016**
Stand and reach (cm)	−1.1 ± 6.4	−1.7 ± 7.6	93	0.268	−0.5 ± 7.5	−1.7 ± 7.6	138	**0.008**	0.379	**0.011**
Push-ups (repetitions)	12.2 ± 3.3	14.2 ± 3.9	97	**<0.001**	13.1 ± 3.9	16.2 ± 4.2	143	**<0.001**	**<0.001**	0.277
Sit-ups (repetitions)	19.4 ± 5.9	20.0 ± 5.2	105	0.217	18.9 ± 6.1	20.9 ± 6.0	160	**<0.001**	**<0.001**	**0.047**
Standing long jump (cm)	128.4 ± 21.7	137.8 ± 23.8	106	**<0.001**	128.8 ± 24.7	138.0 ± 24.7	164	**<0.001**	**<0.001**	0.909
Ergometry (W/kg)	3.1 ± 0.7	3.0 ± 0.7	112	0.099	3.1 ± 0.7	3.2 ± 0.8	173	**<0.001**	**0.031**	**<0.001**

Abbreviations: EG, exercise group; SD, standard deviation; v0, baseline visit; v1, follow-up visit; WG, waiting group. *p*-values < 0.05 are bolded.

**Table 3:** Summary of interaction analyses on the effect of sex, age group, and BMI category on the change in motor skills.

	Sex		Age Group		BMI Category	
	WG	EG	WG	EG	WG	EG
Sprint (Δ s)	ns	ns	**2nd > 5th**	ns	ns	ns
Backwards Balance (Δ steps)	ns	ns	ns	**2nd > 5th**	ns	ns
Side Jumps (Δ repetitions)	ns	ns	ns	ns	ns	ns
Stand and reach (Δ cm)	ns	ns	ns	ns	ns	ns
Push-ups (Δ repetitions)	**m > f**	ns	**2nd > 5th**	**2nd > 5th**	ns	ns
Sit-ups (Δ repetitions)	ns	**f > m**	ns	ns	ns	ns
Standing long jump (Δ cm)	ns	ns	ns	ns	ns	**NW > OW**
Ergometry (Δ W/kg)	ns	ns	ns	**2nd > 5th**	ns	ns

A separate model was calculated for every endpoint. The dependent variable is the change in the respective motor skill after one year. The complete models are shown in Table S4a–h. Abbreviations: BMI, body-mass index; EG, exercise group; f, female; m, male; NW, non-overweight; OW, overweight WG, waiting group. *p*-values < 0.05 are bolded.

## Data Availability

Data can be made available to researchers upon reasonable request.

## Supplementary Materials

The following supporting information can be downloaded at: https://www.mdpi.com/article/10.3390/ijerph20064764/s1.
