# Supplementary material for: Participating in a School-Integrated Daily Exercise Program Improves Motor Performance Significantly in School-Children
Source: Int J Environ Res Public Health. 2023 Mar 8;20(6):4764. doi: 10.3390/ijerph20064764 (PMC10048861; doi:10.3390/ijerph20064764)
Supplement: Supplementary file 1 [file ijerph-20-04764-s001.zip › ijerph-2186265-supplementary.pdf]

Supplemental

Description of each test item of the motor skill test

The 20-meter sprint

Task: Sprinting as fast as possible on a 20 meter distance.

Execution: Two attempts with an acoustic start signal. The timing was done on the finish line with an accuracy of 1/100 seconds with a stopwatch.

Scoring: The better value of two attempts.

Backwards balance

Task: balancing backwards over three beams of different widths consecutively.

Execution: balancing backwards over three beams (6 cm, 4.5 cm and 3 cm) from a starting board, with two attempts. One trial backward and one trial forward were performed in advance for each beam. The test was completed as soon as one foot touched the floor, eight steps were performed without error or the end of the beam was reached.

Scoring: Number of steps achieved, sum of two attempts.

Side Jumps

Task: Side Jumps between two adjacent 50x50 cm fields with both legs, as fast as possible.

Execution: 5 trial jumps. Then two test attempts of 15 seconds with a one-minute break in-between.

Scoring: The number of correctly executed jumps during 15 seconds.

Stand and reach

Task: Bending the upper body down as far as possible without bending the knees.

Execution: Standing on a box (without shoes) to which a centimeter scale was attached so that the sole level represented the zero point. With legs extended, the upper body was brought down until the maximum stretch position reached that could be held for two seconds.

No test trial. Two test attempts, briefly straightening up between them.

Scoring: The lowest point reached with the finger from two attempts.

Push-ups

Task: As many push-ups as possible within 40 seconds.

Execution: Start was lying on a mat in prone position with hands touching on the back. At the start signal, hands were placed next to the shoulders and the body was pushed off the floor until the arms were fully extended. Then one hand was released from the floor and touched the other hand. Both torso and legs were extended and

did not touch the floor. The attempt ended when the starting position was resumed. Two trial attempts, one test run.
Scoring: The number of correctly performed attempts.

Standing-Long Jump

Task: Jumping as far as possible from a standing position with both legs.
Execution: Jumping and landing as far as possible with both legs from a standing position. Two valid attempts, no trial attempts.
Scoring: The distance from the back heel to the take-off line, in centimeters.

Sit-ups

Task: As many sit-ups as possible within 40 seconds.
Execution: Starting lying on a mat in supine position, with feet up and legs bent (about 80°). The fingers held at the temples, thumbs behind the earlobes. At the start signal, the upper body was lifted from the mat. Then the upper body was placed back on the mat only up to the shoulders. Two trial attempts.
Scoring: The number of correctly performed sit-ups.

Ergometry

Task: Riding on an ergometer with increasing resistance until exhaustion.
Execution: The Godfrey protocol (1) was used with submaximal load until exhaustion. The wattage to be pedaled was determined according to the subject's height (<1.20m= 10 Watt, 1.20m - 1.50m= 15 Watt, >1.50m=20 Watt) and increased by 0.5 Watt/kg body weight every two minutes The test was terminated when the subjective exercise limit was reached, or the minimum speed of 50 rpm could not be pedaled for more than 20 seconds. After the termination, three more minutes were run at low resistance.
Scoring: The maximum wattage per kg body weight.

53 ***Short explanations of the movement impulses during class***

Strength games

The sudden squat	Running on the spot and squatting each time a prearranged sound can be heard during a song.
The tree	Two children standing opposite each other, with a third child in the middle as a "tree", swinging back and forth with a stable posture.
Strength circle	Performing various strength exercises (such as lunges, squats, cycling while sitting) for 30 seconds.
Windmill	Circling the arms like a windmill in different speeds and scope, according to the game leader’s specified wind force.
Movement series	Executing a specific strength exercise (such as squats, pushups, planks) according to the previously allocated numbers.
Strength cap	Performing already known strength exercises (such as a lunges, deep sidesquat) with a paper between two children as a connecting point.
The wrong story	Listening to a story in the deep squat position and jumping up once when a false statement occurs in the story.
Sing my song	Singing a song together, a strength exercise (such as push-ups, burpees) is performed each time a pre-determined word of the song text occurs.
Higher / lower	Jumping up if the result is higher than the number written on the board and doing a push-up if the result is lower.
Divisible	Counting in turn. If the number is divisible by a pre-determined number, a lunge is performed.

Endurance games

Fidget time	Fidgeting with the body as strong as possible, freezing at a predefined signal and starting to fidget again.
The hot seat	Performing an endurance task (such as jumping jacks, knee lever ups) sitting on the chair and jumping up after the game leader says *hot* and performing the task in a standing position.
Fruit salad	Kneeling on the spot and starting to jump, when the individual predetermined fruit is mentioned until another fruit is mentioned.
Packing a suitcase	Running on a spot and completing the sentence "I'm packing my suitcase and take with me..." accompanied by a suitable movement. The next child has to repeat and pack another thing.
Magnetic floor	Walking/jumping through the classroom, the children have to bring the given body parts as quickly as possible to the floor after the signal "magnetic floor".

Dashing up and down the stairs	Running up and down the stairs in various alternatives (such as running sideways, skipping a step, jumping) given by the game leader.
Visit to the zoo	Walking through the classroom or running on the spot, the game leader names animals to be imitated in their movements.
30 seconds game	Performing various endurance exercises for 30 seconds (such as jumping jacks, kneeling, alternating jumps)
Fire, Water, Storm	Performing jumping jacks and reacting with a predefined exercise such as running on the spot (fire), swimming movement (water) and planking (storm) to every signal word.
ABC	Reciting the alphabet together and starting to circle the arms, when the letter of your surname is mentioned and changing to kneeling when the letter of the first name is mentioned.

Coordination games

Paddleboat	Performing a pedaling movement with the legs in a sitting position as if sitting in a paddleboat with signals for faster and slower movements, changes of direction and additional arm movements.
The opposite game	Running on the spot and performing the opposite movement the game leader demonstrates while the game leader demonstrates (arms up/down, right/left).
Balancing act	Balancing a ruler on the hand/finger and walking around the classroom. Editing further movement tasks after the initial phase (such as standing on one leg, with closed eyes, moving the other arm up and down).
Corkscrew	Throwing a cork into the hand of a partner. Once this task is well mastered, the difficulty is increased (standing on one leg, clapping hands while the cork flies, running on the spot, etc.)
Zig Zag Ball	Throwing a small ball in a certain order, then a second ball with another certain order is added.
Move it	Performing the following movements in sequence with the focus on coordination, with variable tempo and direction: jumping on the spot 5 times, jumping forth and back, jumping sideways 2 times, jumping in a circle 2 times, small straddle, small lunge, final pose.
Moving memory	Children form pairs. Each pair secretly agrees on a coordinative exercise (such as jumping jacks, left hand to the right foot and the other way around.). Children are then selected one at a time to perform this exercise, until all pairs have been found.
Circling arms	Circling the arms according to the given tempo and direction with additional tasks like jumping on one leg.

Simon says	Drumming with the fingers on the table, the game leader calls coordinative exercises (such as standing on one leg, circling arms in opposite directions) which are performed only, if command "Simon says" is mentioned before.
Jumping leg	Jumping according to the given commands: right leg twice and left leg once, sideways, forward or backwards, on one leg fast/slow etc.

Relaxation games

Raindrops	Lightly tapping with the fingers over different parts of the body as if raindrops were touching them.
The moving shadow	Imitating the slow, large and smooth movements of a partner, breathing slow and deep.
Ball massage	Massaging the hands by circling a small ball with one hand on the table and afterwards the feet, thighs and arms.
Stretch and strain	Stretching: Trying to be as big as possible, strain: stretching as it is comfortable, circles: circle the hips, hands and head, tease: make faces
Pizza game	Imitating the process of pizza baking on the back of a partner: Stroking the back with the hands, smearing the sauce on the dough, etc.
Animal Yoga	Imitating animals with slow movements such as slow and big steps like an elephant accompanied with deep breathing.
Growing flower	Imitating the growth process of a flower through the seasons, from a small stooped posture to a stretching position.
Relaxed eyes	Placing warm hands rubbed together in advance on the closed eyes, breathing deeply in and out
Stroking	Stroking on the legs, the arms, the belly and face. Slow, lightly accompanied with deep breathing.
Make a fist	In a seated position making and opening a fist and concentrating to the feeling of Tension and relaxation.

Variations of movements are suggested to the teachers on the index cards.

54

Figure S1. Examples of Index cards used in class

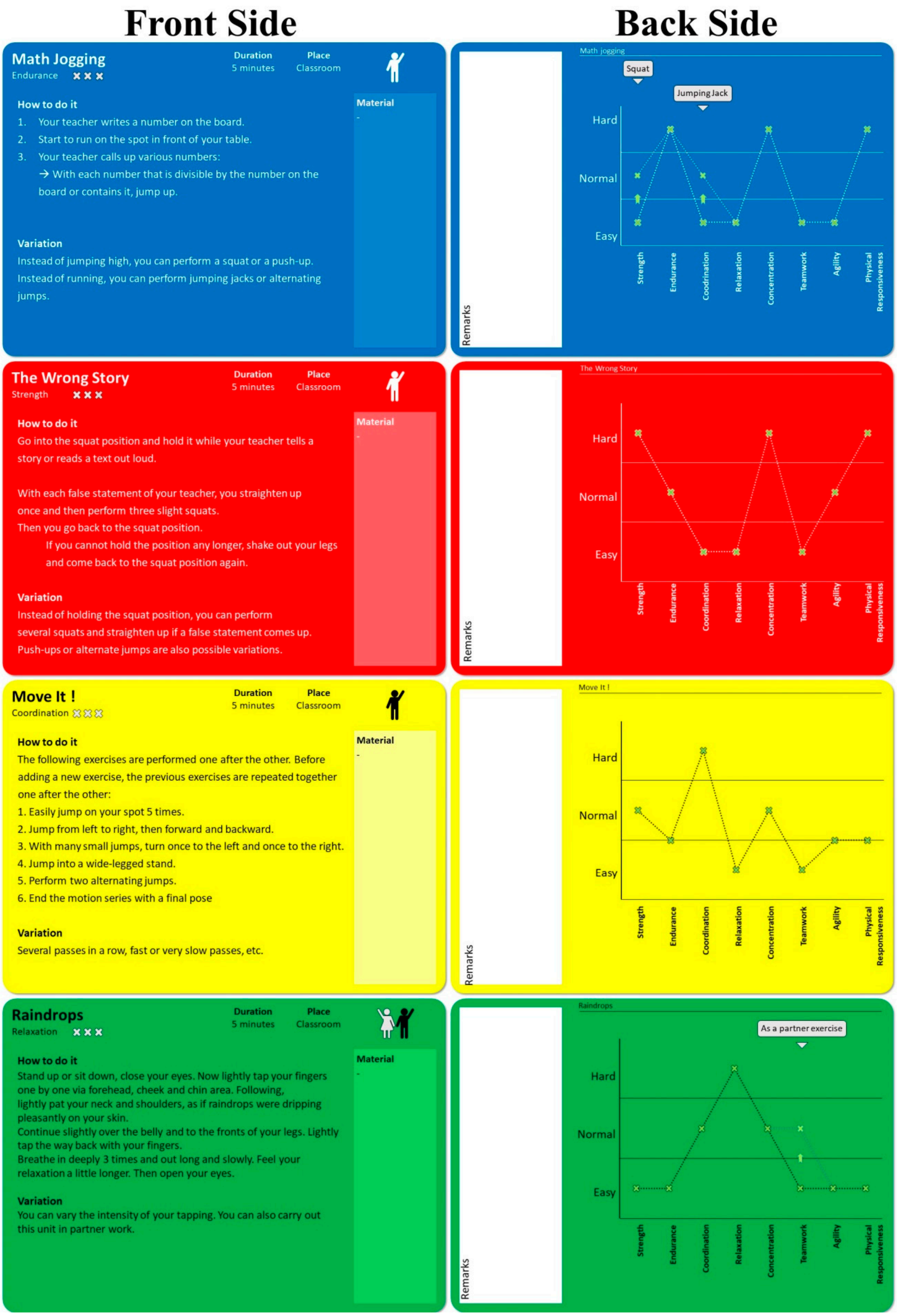

Figure S2. Flow diagram

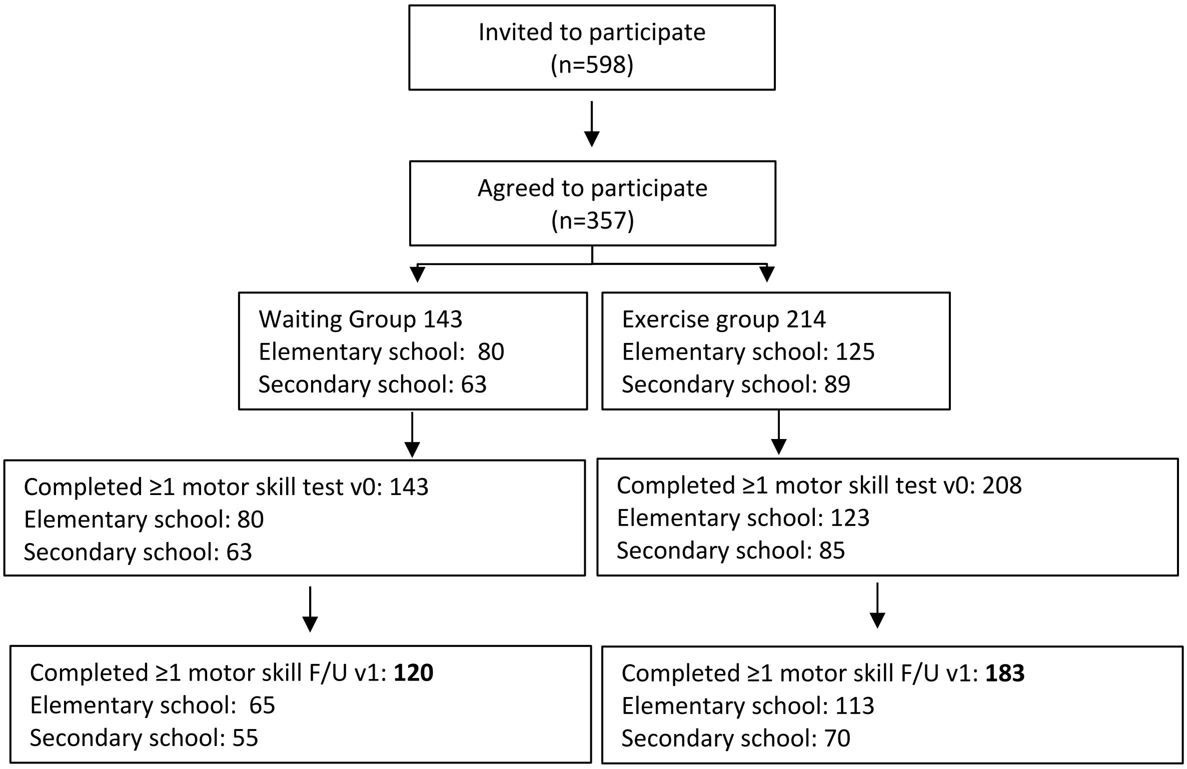

v0= Baseline examination, v1=Follow-up examination

Table S1 Results of the motor skills at baseline, stratified by sex

	Boys		Girls		
	Mean ± SD	n	Mean ± SD	n	p
Sprint (sec)	4.4±0.5	148	4.6±0.4	134	**<0.001**
Backwards Balance (steps)	29.3±9.9	140	28.8±10.3	121	0.663
Side Jumps (jumps)	26.3±7.2	143	25.5±6.8	129	0.349
Stand and reach (cm)	-2.9±6.2	131	1.9±7.0	110	**<0.001**
Push-ups (repetitions)	12.7±4.0	138	12.8±3.3	117	0.823
Sit-ups (repetitions)	19.8±6.4	148	17.9±5.7	133	**0.011**
Standing long jump (cm)	134.5±26.4	148	120.6±19.6	134	**<0.001**
Ergometry (W/kg)	3.3±0.7	155	2.9±0.5	140	**<0.001**

Abbreviations: WG, Waiting group; EG, Exercise group; SD, Standard deviation. $p<0.05$ are bolded.

Table S2 Results of the motor skills at baseline, stratified by age group

	2nd grade		5th grade		
	Mean ± SD	n	Mean ± SD	n	p
Sprint (sec)	4.6±0.4	169	4.4±0.5	113	**<0.001**
Backwards Balance (steps)	28.5±10.0	148	29.8±10.1	113	0.327
Side Jumps (jumps)	23.6±6.1	159	29.2±7.0	113	**<0.001**
Stand and reach (cm)	1.6±3.4	127	-3.3±6.8	114	**<0.001**
Push-ups (repetitions)	12.6±3.5	141	12.9±3.9	114	0.500
Sit-ups (repetitions)	18.5±6.0	167	19.4±6.3	114	0.424
Standing long jump (cm)	122.4±22.5	169	136.2±24.8	113	**<0.001**
Ergometry (W/kg)	3.1±0.7	175	3.1±0.7	120	0.402

Abbreviations: WG, Waiting group; EG, Exercise group; SD, Standard deviation. $p<0.05$ are bolded.

Table S3 Results of the motor skills at baseline, stratified by BMI category

	Non-Overweight		Overweight		p
	Mean ± SD	n	Mean ± SD	n	
Sprint (sec)	4.5±0.4	207	4.7±0.5	74	**<0.001**
Backwards Balance (steps)	30.9±9.6	193	23.9±9.8	67	**<0.001**
Side Jumps (jumps)	26.6±7.1	197	24.1±6.8	74	**0.009**
Stand and reach (cm)	-0.5±6.8	175	-1.4±7.5	65	0.372
Push-ups (repetitions)	13.3±3.6	187	11.1±3.4	67	**<0.001**
Sit-ups (repetitions)	19.7±5.7	208	16.5±6.7	72	**<0.001**
Standing long jump (cm)	132.1±23.9	207	116.1±22.0	74	**<0.001**
Ergometry (W/kg)	3.3±0.6	216	2.5±0.5	79	**<0.001**

Abbreviations: WG, Waiting group; EG, Exercise group; SD, Standard deviation. p<0.05 are bolded.

**Table S4a** Change in the sprint test (Δ sec), n=272			
	β	SE	p
Intercept	-0.325	0.067	<0.001
EG (Ref: WG)	0.084	0.086	0.329
WG*female (Ref: WG*male)	0.065	0.080	0.4171
EG*female (Ref: EG*male)	-0.059	0.062	0.345
**WG*5th grade (Ref: WG*2nd grade)**	**0.347**	**0.080**	**<0.001**
EG*5th grade (Ref: EG*2nd grade)	-0.096	0.065	0.137
WG*overweight (Ref: WG*non-overweight)	-0.069	0.090	0.445
EG*overweight (Ref: EG*non-overweight)	0.084	0.073	0.246
Abbreviations: WG, Waiting group; EG, Exercise group; β Regression Coefficient; SE, Standard Error; p<0.05 are bolded.			

**Table S4b** Improvement in Balance (Δ steps), n=250			
	β	SE	p
Intercept	2.715	1.524	0.076
**EG (Ref: WG)**	**5.308**	**1.927**	**0.006**
WG*female (Ref: WG*male)	3.532	1.796	0.0504
EG*female (Ref: EG*male)	-0.272	1.388	0.845
WG*5th grade (Ref: WG*2nd grade)	-0.311	1.774	0.861
**EG*5th grade (Ref: EG*2nd grade)**	**-4.934**	**1.426**	**<0.001**
WG*overweight (Ref: WG*non-overweight)	-2.707	1.978	0.173
EG*overweight (Ref: EG*non-overweight)	0.163	1.655	0.922
Abbreviations: WG, Waiting group; EG, Exercise group; β Regression Coefficient; SE, Standard Error; p<0.05 are bolded.			

**Table S4c** Improvement in Side Jump (Δ jumps), n=256			
	β	SE	p
Intercept	4.250	1.003	<0.001
EG (Ref: WG)	1.047	1.290	0.418
WG*female (Ref: WG*male)	-0.885	1.176	0.452
EG*female (Ref: EG*male)	0.671	0.943	0.477
WG*5th grade (Ref: WG*2nd grade)	-0.870	1.174	0.459
EG*5th grade (Ref: EG*2nd grade)	-0.218	0.965	0.822
WG*overweight (Ref: WG*non-overweight)	-0.145	1.310	0.912
EG*overweight (Ref: EG*non-overweight)	-0.829	1.070	0.439
Abbreviations: WG, Waiting group; EG, Exercise group; β Regression Coefficient; SE, Standard Error; p<0.05 are bolded.			

**Table S4d** Improvement in Stand and Reach (Δ cm), n=230			
	β	SE	p
Intercept	-1.261	0.966	0.193
EG (Ref: WG)	1.584	1.250	0.206
WG*female (Ref: WG*male)	0.620	1.120	0.581
EG*female (Ref: EG*male)	0.761	0.902	0.399
WG*5th grade (Ref: WG*2nd grade)	0.852	1.105	0.442
EG*5th grade (Ref: EG*2nd grade)	0.784	0.910	0.390
WG*overweight (Ref: WG*non-overweight)	-0.117	1.232	0.924
EG*overweight (Ref: EG*non-overweight)	0.632	1.029	0.540
Abbreviations: WG, Waiting group; EG, Exercise group; β Regression Coefficient; SE, Standard Error; p<0.05 are bolded.			

Table S4e Improvement in Push-ups (Δ push-ups), n=239

	β	SE	p
Intercept	4.259	0.685	<0.001
EG (Ref: WG)	-0.992	0.883	0.263
**WG*female (Ref: WG*male)**	**-1.826**	**0.802**	**0.024**
EG*female (Ref: EG*male)	0.914	0.648	0.160
**WG*5th grade (Ref: WG*2nd grade)**	**-1.879**	**0.793**	**0.019**
**EG*5th grade (Ref: EG*2nd grade)**	**-1.405**	**0.658**	**0.034**
WG*overweight (Ref: WG*non-overweight)	-0.115	0.898	0.898
EG*overweight (Ref: EG*non-overweight)	0.091	0.742	0.903

Abbreviations: WG, Waiting group; EG, Exercise group; β Regression Coefficient; SE, Standard Error; p<0.05 are bolded.

Table S4f Improvement in Sit-ups (Δ sit-ups), n=264

	β	SE	p
Intercept	1.714	0.867	0.049
EG (Ref: WG)	-0.171	1.125	0.880
WG*female (Ref: WG*male)	-1.834	1.047	0.081
**EG*female (Ref: EG*male)**	**1.862**	**0.835**	**0.027**
WG*5th grade (Ref: WG*2nd grade)	-0.940	1.047	0.370
EG*5th grade (Ref: EG*2nd grade)	-0.838	0.862	0.332
WG*overweight (Ref: WG*non-overweight)	0.168	1.208	0.889
EG*overweight (Ref: EG*non-overweight)	-0.676	0.967	0.485

Abbreviations: WG, Waiting group; EG, Exercise group; β Regression Coefficient; SE, Standard Error; p<0.05 are bolded.

Table S4g Improvement in Standing Long Jumps (Δ jumps), n=269

	β	SE	p
Intercept	12.931	2.719	<0.001
EG (Ref: WG)	-2.069	3.524	0.558
WG*female (Ref: WG*male)	-3.868	3.274	0.239
EG*female (Ref: EG*male)	3.466	2.599	0.183
WG*5th grade (Ref: WG*2nd grade)	-2.434	3.276	0.458
EG*5th grade (Ref: EG*2nd grade)	-4.484	2.693	0.097
WG*overweight (Ref: WG*non-overweight)	-3.800	3.694	0.305
**EG*overweight (Ref: EG*non-overweight)**	**-6.864**	**3.008**	**0.023**

Abbreviations: WG, Waiting group; EG, Exercise group; β Regression Coefficient; SE, Standard Error; $p<0.05$ are bolded.

Table S4h Improvement in Ergometry (ΔWatt/kg), n=268

	β	SE	p
Intercept	0.002	0.065	0.981
**EG (Ref: WG)**	**0.322**	**0.084**	**<0.001**
WG*female (Ref: WG*male)	-0.052	0.077	0.502
EG*female (Ref: EG*male)	-0.083	0.061	0.176
WG*5th grade (Ref: WG*2nd grade)	0.018	0.077	0.816
**EG*5th grade (Ref: EG*2nd grade)**	**-0.216**	**0.064**	**<0.001**
WG*overweight (Ref: WG*non-overweight)	-0.111	0.086	0.202
EG*overweight (Ref: EG*non-overweight)	-0.119	0.070	0.089

Abbreviations: WG, Waiting group; EG, Exercise group; β Regression Coefficient; SE, Standard Error; $p<0.05$ are bolded.

Supplement References

(1) Godfrey S, Davies CT, Wozniak E, Barnes CA. Cardio-respiratory response to exercise in normal children. Clin Sci 1971 May;40(5):419-431.
